# Anæsthesia in Surgery.—Who Discovered It?

**Published:** 1870-04

**Authors:** 


### ANÆSTHESIA IN SURGERY.—WHO DISCOVERED IT?

Sir J. Y. Simpson, Professor of Obstetrics in the Edinburgh University, in a published letter, has recently called special attention to this question. Dr. Simpson has investigated the evidence, and does not hesitate to award the merit to Dr. Horace Wells of this city, who inhaled nitrous oxide gas on the 11th of December, 1844, for the purpose of having a tooth pulled under its influence. Dr. Simpson, however, assumes that Sir Humphrey Davy used this gas to deaden pain at the close of the last century; that it was used in the 15th century; and also in about the year 300, for the same purpose. But Dr. Simpson does not claim, nor does he attempt to prove, that it was introduced to the world and acknowledged, or even known to the surgeons as an anæsthetic agent in surgical operations, till Dr. Wells, with the assistance of John M. Riggs, introduced it, and proved its success, on the 11th day of December, 1844. The history of the discovery, and the practical test, at that time, is as follows:

### EXHIBITION OF LAUGHING GAS.

On the 10th of December, 1844, Dr. G. Q. Colton gave an exhibition in Hartford of the effects of Nitrous Oxide or "Laughing Gas." He did not attempt to produce complete anæsthesia, but administered the gas till the subject became exhilarated, so as to act, in a partially unconscious state, the leading characteristics of his nature. Some of his subjects laughed, some cried, others tried to fight, and some prayed, or made demonstrations of the passion of love, hate or some other characteristic. Mr. Colton's object was to make money. He had no idea that his exhibition was to develop and bring into practical use an agent of the greatest value to science, and of inestimable blessings to mankind. We here subjoin the complete advertisement of Dr. Colton. It is copied from the Hartford *Daily Times*, of Dec. 10, 1844:

"ADVERTISEMENT—'LAUGHING GAS."

"A GRAND EXHIBITION of the effects produced by inhaling Nitrous Oxide, Exhilarating or Laughing Gas! will be given in Union Hall on *Tuesday evening, December* 10, 1844. Forty gallons of gas will be prepared and administered to all in the audience who desire to inhale it. Twelve young men have volunteered to inhale the gas, to commence the entertainment. Eight strong men are engaged to occupy the front seats, to prevent those under the influence of the gas from injuring themselves or others. This course is adopted, that no apprehension of danger may be entertained. Probably no one will attempt to fight.

"The effect of the gas is to make those who inhale it either laugh, sing, dance, speak or fight, etc., according to the leading trait of their character. They seem to retain consciousness enough not to say or do that which they would have occasion to regret. N. B.—The gas will be administered only to gentlemen of the first respectability. The object is to make the entertainment in every respect a genteel affair.

"Mr. Colton, who offers this entertainment, gave two of the same character last spring in the Broadway Tabernacle, New York, which were attended by over four thousand ladies and gentlemen, a full account of which may be found in the New York *Mirror* of the 6th, N. P. Willis. Being on a visit to Hartford he offers this entertainment at the earnest solicitation of friends. It is his wish and intention to *deserve* and receive the patronage of the first class. He believes he can make them laugh more than they have for the six months previous. The entertainment is *scientific* to those who make it scientific. Those who inhale the gas once are always anxious to inhale it the second time. There is not an exception to this rule. No language can describe the delightful sensation produced. Robert Southey (poet) once said that the "atmosphere of the highest of all possible heavens must be composed of this gas.' For a full account of the effect produced upon some of the most distinguished men of Europe, see Hooper's Medical Dictionary, under the head of Nitrogen.

"Mr. Colton will be the first to inhale the gas. The history and properties of the gas will be explained at the commencement of the entertainment, which will close with a few of the most surprising chemical experiments.

"Mr. Colton will give a private entertainment to those ladies who desire to inhale the gas, on Tuesday, between 12 and 1 o'clock, free. None but ladies will be admitted. This is *intended* for those who desire to inhale the gas, though others will be admitted.

"Entertainment to commence at 7 o'clock. Tickets 25 cents. For sale at the principal bookstores, and at the door. [dec. 9—2d.]"

We readily found this advertisement in the files of the *Times*, from the memorandum of Dr. Riggs recording the experiment upon Dr. Colton's exhibition.

Drs. Wells and Riggs, well known surgeon dentists of this city, attended this exhibition to see the fun which might come from a display of the latent but concealed characteristics and passions of those of our citizens who might be induced to inhale the "Laughing Gas."

Among those who inhaled the gas, during this exhibition, was Col. S. A. Cooley of this city, who, as soon as the exhilarating effects came over him, dashed through the "eight strong men on the front seats, engaged to prevent those who inhaled the gas from injuring themselves or others," and he flew about the room at a furious rate. Over the benches, back and forth he rushed, tearing his flesh and bruising his legs and arms in a painful manner. When he recovered from the influence of the gas he was well marked by the injuries he had received. Dr. Wells went to him, and earnestly enquired if those thumps and cuts did not hurt him? "Not in the least—I did not feel them," replied Col. Cooley. "Then," said Dr. Wells, "I can pull a tooth under the influence of that gas without hurting the patient." The great idea had flashed upon his mind.

### THE SURGEONS IN CONSULTATION.

Drs. Wells and Riggs left the hall together, and repaired to the operating room of the latter. It was no longer a "laughing" but a very serious question. Could this gas be used in surgical operations? Would it produce complete anæsthesia, and deaden all sense of pain? Was it safe? or would its use endanger the lives of patients? These were the questions discussed by the two surgeon dentists till midnight, on that same eventful 10th of December, 1844. Dr. Riggs, who was a graduate at college, gave the history of experiments which he had seen in the laboratory with nitrous oxide gas. He had never seen nor heard of surgical operations under its influence; but when a college student he had witnessed experiments which satisfied him that it would produce complete anæsthesia, and that it could be safely administered. Dr. Wells alluded to the precautions of Dr.

Colton, who had hired eight men to hold his subjects when under the influence of the gas; and what, he asked, are we to do with patients in our chairs, who had teeth to be pulled? They will jump up, fight, dash out of the window, perhaps, and possibly some of them will kill themselves. It was suggested that they be tied with strong cords. But that would not do; that strong men should hold them, but that hardly appeared practicable. Dr. Riggs still insisted that he had seen enough in the college laboratory to satisfy him that the gas would put the patient into such a sound sleep that he would neither fight, stir, nor do harm.

Who, then, was to be the first subject? And here, for a moment, both of the surgeons shrunk from the experiment. Suppose we take the first one that comes? Suppose he *dies* under the operation? Then, they concluded, both of them would be hanged, or sent to State prison for life. Still, the idea that had flashed across the mind of Dr. Wells on witnessing the injuries to Col. Cooley without producing pain, had roused all his ambition, hopes, and determination; and Dr. Riggs felt no less interest in the subject than Dr. Wells. The two had ranged over the whole field of probabilities and dangers, and had discussed the momentous results of the discovery, in case it should really prove safe and a success. It was now midnight, and something was to be done—an experiment was to be tried—speculation and argument had done all that they could accomplish.

### A TEST—A TOOTH TO BE PULLED.

"I have a tooth," said Dr. Wells, "a little decayed, sufficient to be filled, *and it shall be pulled!* We will prepare the gas. I will take it, and *you shall pull the tooth to-morrow!*" said he to Dr. Riggs, who assented to this proposition, and said, "it is fair to begin on ourselves"—and on that, the surgeons separated for the night, with fixed determination and high hopes.

In the morning, Dec. 11, 1844, the gas was prepared. At half-past ten o'clock A. M., Dr. Wells took the dentist's chair in his own office, at the corner of Main and Asylum streets—the office now occupied by Dr. Taft. Half a dozen friends were invited in. All of them laughed at the wild notions of the surgeons—none of them believed in the success of the experiment about to be tried. But Dr. Wells took the pipe of the gas-bag in his mouth, and it was kept there till his hands fell lifeless, and his head drooped. Then in an instant Dr. Riggs fastened his forceps to the left upper molar, and drew it out. Dr. Wells did not move a muscle. Holding the tooth up to the spectators, Dr. Riggs said, "You see!"

In a moment Dr. Wells opened his eyes, blew the blood from his mouth, and asked, "Is my tooth out?"

"It is," said Dr. Riggs.

"A new era in tooth pulling!" exclaimed Dr. Wells, clapping his hands in great joy, and declaring that he did not feel any pain when the tooth was pulled.

Dr. Marcy, a practicing physician and surgeon in this city (now of New York), hearing of this great triumph, got Dr. Wells to assist him, a few days later, and in the amputation of a leg from a boy in East Hartford, used the gas, and cut off the limb, without inflicting any pain. A few days after that Dr. Marcy removed a tumor under the gas influence, also without pain—and the success of anæsthesia in surgery was practically established. Doctors Wells and Riggs pulled teeth, successfully using the gas, time and again—and from their experiment on the 11th of December, 1844, was spread over the world, to the relief of untold agony, the great discovery, now used universally in surgery, obstetrics, and indeed in all the phases of surgery. Dr. Morton, a student of Dr. Wells, tried a year or two later, to rob Dr. Wells of the discovery, but failed in the undertaking.

